# Production of (*S*)-2-aminobutyric acid and (*S*)-2-aminobutanol in *Saccharomyces cerevisiae*

**DOI:** 10.1186/s12934-017-0667-z

**Published:** 2017-03-23

**Authors:** Nora Weber, Anaëlle Hatsch, Ludivine Labagnere, Harald Heider

**Affiliations:** Evolva SA, Duggingerstrasse 23, 4153 Reinach, Switzerland

**Keywords:** (*S*)-2-Aminobutyric acid, l-Homoalanine, (*S*)-2-Aminobutanol, Carboxylic acid reductase, l-Threonine, 2-Ketobutyric acid, Metabolic engineering, Ethambutol

## Abstract

**Background:**

*Saccharomyces cerevisiae* (baker’s yeast) has great potential as a whole-cell biocatalyst for multistep synthesis of various organic molecules. To date, however, few examples exist in the literature of the successful biosynthetic production of chemical compounds, in yeast, that do not exist in nature. Considering that more than 30% of all drugs on the market are purely chemical compounds, often produced by harsh synthetic chemistry or with very low yields, novel and environmentally sound production routes are highly desirable. Here, we explore the biosynthetic production of enantiomeric precursors of the anti-tuberculosis and anti-epilepsy drugs ethambutol, brivaracetam, and levetiracetam. To this end, we have generated heterologous biosynthetic pathways leading to the production of (*S*)-2-aminobutyric acid (ABA) and (*S*)-2-aminobutanol in baker’s yeast.

**Results:**

We first designed a two-step heterologous pathway, starting with the endogenous amino acid l-threonine and leading to the production of enantiopure (*S*)-2-aminobutyric acid. The combination of *Bacillus subtilis* threonine deaminase and a mutated *Escherichia coli* glutamate dehydrogenase resulted in the intracellular accumulation of 0.40 mg/L of (*S*)-2-aminobutyric acid. The combination of a threonine deaminase from *Solanum lycopersicum* (tomato) with two copies of mutated glutamate dehydrogenase from *E. coli* resulted in the accumulation of comparable amounts of (*S*)-2-aminobutyric acid. Additional l-threonine feeding elevated (*S*)-2-aminobutyric acid production to more than 1.70 mg/L. Removing feedback inhibition of aspartate kinase HOM3, an enzyme involved in threonine biosynthesis in yeast, elevated (*S*)-2-aminobutyric acid biosynthesis to above 0.49 mg/L in cultures not receiving additional l-threonine. We ultimately extended the pathway from (*S*)-2-aminobutyric acid to (*S*)-2-aminobutanol by introducing two reductases and a phosphopantetheinyl transferase. The engineered strains produced up to 1.10 mg/L (*S*)-2-aminobutanol.

**Conclusions:**

Our results demonstrate the biosynthesis of (*S*)-2-aminobutyric acid and (*S*)-2-aminobutanol in yeast. To our knowledge this is the first time that the purely synthetic compound (*S*)-2-aminobutanol has been produced in vivo. This work paves the way to greener and more sustainable production of chemical entities hitherto inaccessible to synthetic biology.

**Electronic supplementary material:**

The online version of this article (doi:10.1186/s12934-017-0667-z) contains supplementary material, which is available to authorized users.

## Background

(*S*)-2-Aminobutyric acid (ABA), also known as l-homoalanine, is a non-proteinogenic α-amino acid, which is a chiral precursor for the enantiomeric pharmaceuticals levetiracetam, brivaracetam, and ethambutol [1, 2]. Enzymatic synthesis of chiral (*R*)- or (*S*)-2-aminobutyric acid has been reached by resolution of *racemic* mixtures with acylase [3, 4] or amidases [5], and asymmetric synthesis starting from 2-ketobutyric acid has been achieved using ω-transaminases [6, 7] or amino acid dehydrogenases [8]. Both (*R*)- and (*S*)-2-aminobutyric acid were also synthesized using an (*R*)- or (*S*)-selective ω-transaminase and isopropylamine as co-substrate [9]. Biosynthesis of (*S*)-2-aminobutyric acid (starting from the amino acid l-methionine) has been shown by co-immobilization of l-methionase and glutamate dehydrogenase on polyacrylamide or chitosan [1].

In contrast to in vitro synthesis with purified enzymes, whole-cell biocatalysts offer the advantage of simple and economical upstream processing as overheads for cell lysis, purification, and addition of co-factors are redundant. Low cost media and straightforward downstream processes further contribute to a more cost-effective production process. In vivo synthesis of (*S*)-2-ABA has been achieved via two different methods: (1) kinetic resolution of *racemic* 2-aminobutyric acid mixture with d-amino acid oxidase and ω-transaminase [10], and (2) asymmetric synthesis from l-threonine [11]. The latter setup used five *E. coli* strains containing five different genes.

(*S*)-2-aminobutanol is an important chiral building block for the chemical synthesis of the bacteriostatic anti-tuberculosis agent (*S, S*)-ethambutol [12]. Activity assays using *Mycobacterium smegmatis* have shown that the (*S*, *S*)-configured diastereomer of ethambutol [(*S*, *S*)-2,20-(ethylenediimino)-di-butanol] has the highest activity compared to all other measured derivatives, and is about 500 times more potent than the (*R*, *R*)-diastereomer [13]. As ethambutol shows ocular toxicity as a side effect [14], it is desirable to keep the therapeutic dose as low as possible. Therefore, it is crucial to apply the potent (*S*, *S*)-configured diastereomer exclusively. Several approaches have been developed to achieve chemical synthesis of enantiopure (*S, S*) ethambutol; e.g. resolution of *racemic* (*S*)-2-aminobutanol [15, 16]; use of palladium to accomplish regio-and stereo-selective epoxide opening [17]; use of l-methionine as chiral starting material [18, 19]; and introduction of protection groups during synthesis [20, 21].

The application of engineered microorganisms for production of chemical building blocks with very high enantiomeric purity may help to improve the supply with cost-efficient active pharmaceutical compounds and, in contrast to chemical synthesis, no additional steps or protection groups are necessary. Increasingly, metabolic engineering and biotechnology are providing sustainable and cost-efficient production of precursors for a variety of pharmaceuticals [22–25].

To our knowledge, (*S*)-2-aminobutyric acid has not been produced in *Saccharomyces cerevisiae*, and (*S*)-2-aminobutanol has not yet been produced in any engineered microorganism. Here, we have explored the possibilities of using the well-known GRAS microorganism *S. cerevisiae* for biosynthesis of (*S*)-2-aminobutyric acid and subsequent production of (*S*)-2-aminobutanol. We demonstrate the in vivo production, in *S. cerevisiae,* of enantiopure (*S*)-2-aminobutyric acid and (*S*)-2-aminobutanol.

## Results

### Production of (*S*)-2-aminobutyric acid

**Fig. 1:**
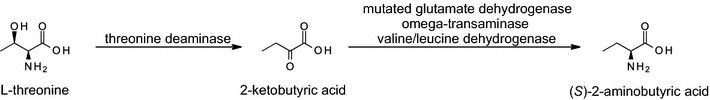
Pathway from l-threonine to (*S*)-2-aminobutyric acid

We have expanded the metabolic capacity of *S. cerevisiae* to generate (*S*)-2-aminobutyric acid ((*S*)-2-ABA) by introducing two heterologous enzymatic steps. Biosynthesis of this non-natural amino acid starts via deamination of l-threonine into the achiral intermediate 2-ketobutyric acid. This keto acid becomes then aminated in a second enzymatic step to yield (*S*)-2-ABA (Fig. 1).

Yeast-endogenous enzymes CHA1 and/or ILV1 in principal can execute this first enzymatic step. Regulation of the corresponding encoding genes occurs in part at the promoter level: to overcome this regulation the coding sequences have been functionally linked to constitutive promoters. An alternative strategy using heterologous enzymes for this first step was also employed. We selected three heterologous threonine deaminases derived from *Escherichia coli*, *Bacillus subtilis*, and *Solanum lycopersicum*.

Based on the results from Zhang and co-workers we selected three mutated glutamate dehydrogenases (two from *S. cerevisiae* and one from *E. coli*) for the second enzymatic step [26]. In addition, we assessed two different leucine dehydrogenases (from *B. cereus* and *B. flexus*), and a valine dehydrogenase (from *S. fradiae*) as putative enzymes for the amination step from 2-ketobutyric acid to (*S*)-2-aminobutyric acid [8, 27].

**Fig. 2:**
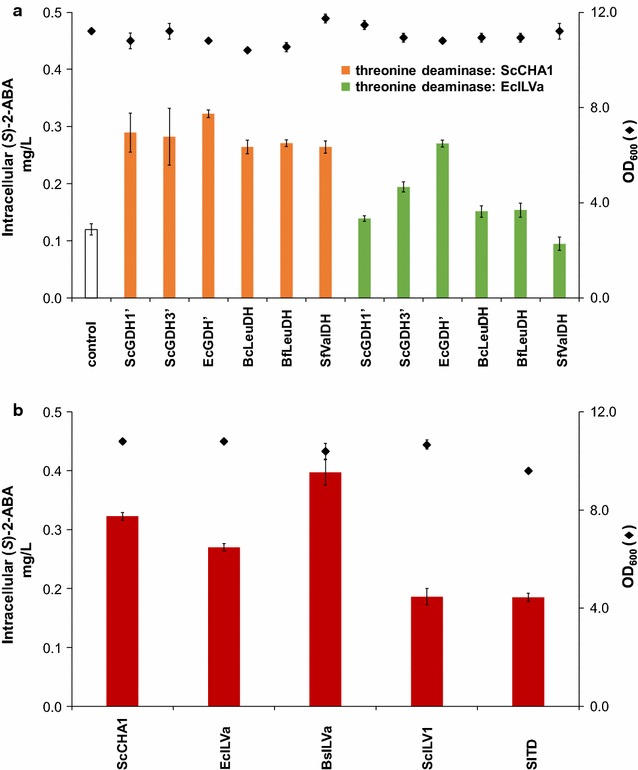
Intracellular accumulation of (*S*)-2-aminobutyric acid in *S. cerevisiae* expressing different enzyme combinations. **a** Either two different threonine deaminases (*orange bars* ScCHA1, *green bars* EcILVa) for the first step of the (*S*)-2-aminobutyric acid pathway were expressed in combination with either one of six heterologous enzymes for the second step of the pathway. Yeasts that did not express the pathway enzymes were included as control. **b** EcGDH’ for the second step of the pathway was expressed in combination with either one of five different threonine deaminases. *Diamonds* indicate OD_600_ after 24 h of growth (all data: mean ± SD, n = 3)

In total 15 *S. cerevisiae* strains were constructed, containing different combinations of threonine deaminases and dehydrogenases. Expressing yeast CHA1 under the control of the constitutively active GPD1 promoter, together with one of three heterologous enzymes for the second step of the pathway, or together with any of the three mutated glutamate dehydrogenases, yielded an accumulation of 0.32 ± 0.01 mg/L (*S*)-2-ABA upon 24 h of culture (Fig. 2a, orange bars). Significant shorter production times led to titers close to the limit of quantification. A longer production time enhanced the amount of (*S*)-2-ABA. For practical reasons a time of 24 h was chosen. Chiral HPLC analysis revealed the resultant (*S*)-2-ABA to be of high enantiopurity (Additional file 1: Figure S1).

A less pronounced increase in (*S*)-2-ABA production was observed when using *E. coli* threonine deaminase (EcILVa) for the first enzymatic step (Fig. 2a, green bars). As the mutated *E. coli* GDH (EcGDH’) yielded the highest concentrations of (*S*)-2-ABA in combination with both ScCHA1 and EcILVa, we chose EcGDH’ to analyze the effect of the threonine deaminases derived from *E. coli*, *B. subtilis*, and *S. lycopersicum* on (*S*)-2-ABA production. Combining ILVa from *B. subtilis* with EcGDH’ led to the highest amount of (*S*)-2-ABA (0.40 ± 0.02 mg/L (*S*)-2-ABA, Fig. 2b). Expression of the heterologous enzymes had negligible effect on strain growth, as reflected by the relatively constant OD_600_ values after 24 h of culture.

### Extension of the heterologous pathway for production of (*S*)-2-aminobutanol

**Fig. 3:**
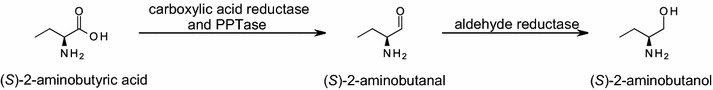
Pathway from (*S*)-2-aminobutyric acid to (*S*)-2-aminobutanol

Enantiopure (*S*)-2-aminobutanol is a building block for the synthesis of the tuberculosis drug ethambutol. The chirality of (*S*)-2-aminobutyric acid is defined by the amine group, which makes it a well-suited intermediate for the biosynthesis of (*S*)-2-aminobutanol. Carboxylic acid reductases (CARs) and aldehyde reductases are enzymes that can perform the final enzymatic steps from (*S*)-2-aminobutyric acid to (*S*)-2-aminobutanol (Fig. 3) by reduction of the terminal carboxylic acid group of (*S*)-2-aminobutyric acid to an alcohol, leaving the stereo center of the molecule intact.

We expressed one of four different carboxylic acid reductases (CARs) in combination with either a phosphopantetheinyl-transferase (PPTase) from *M. smegmatis* (for *M. smegmatis* CAR), or the one from *B. subtilis* (SFP) for all other CARs. CARs, when expressed in yeast, require a PPTase for activity [28]. Expression of a heterologous aldehyde reductase derived from *E. coli* in our strains facilitated reduction of the (*S*)-2-aminobutanal to the corresponding alcohol (Fig. 3). Initial supplementation with additional (*S*)-2-ABA (0.5 g/L) was tested, as we observed rather low production of (*S*)-2-ABA.

**Fig. 4:**
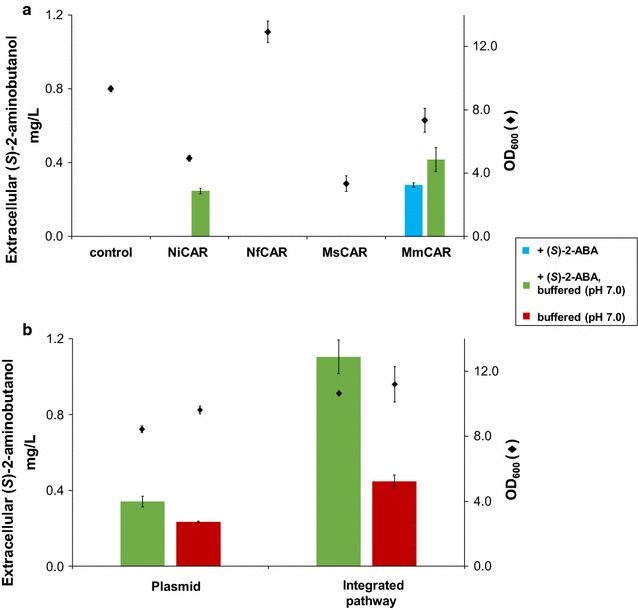
Extracellular accumulation of (*S*)-2-aminobutanol in *S. cerevisiae*. **a** Yeasts were transformed with plasmids harboring the sequences encoding a threonine deaminase and EcGDH’, in combination with different carboxylic acid reductases. PPTases were either from *Mycobacterium smegmatis* (for MsCAR) or from *Bacillus subtilis* (SFP) for all other CARs. The aldehyde reductase for the last step of the pathway was derived from *E. coli*. Engineered yeasts were incubated for 72 h in selective SC medium containing 0.5 g/L (*S*)-2-aminobutyric acid (+(*S*)-2-ABA). The medium was either buffered to pH 7 (*green bars*), or supplied unbuffered (*blue bar*). Supernatants were analyzed for (*S*)-2-aminobutanol production. **b** All five pathway genes (CAR from *Mycobacterium marinum*) were integrated into chromosome XI-2. Yeasts were incubated for 72 h in selective SC medium buffered to pH 7, and were either supplied with 0.5 g/L (*S*)-2-aminobutyric acid (*green bars*), or were grown without additional (*S*)-2-aminobutyric acid supply (*red bars*). *Diamonds* indicate OD_600_ after 72 h of growth (all data: mean ± SD, n = 3)

LC–MS analysis of pellets and supernatants from cells grown for 72 h in non-buffered conditions in shake flasks revealed quantifiable amounts of the target compound (*S*)-2-aminobutanol exclusively in the supernatant, and only in strains expressing the carboxylic acid reductase from *Mycobacterium marinum* (Fig. 4a, blue bar). Shorter production times led to titers of (*S*)-2-aminobutanol below the limit of quantification.

Adjusting the culture medium to pH 7 boosted production levels. The neutral pH resulted in a significant increase of (*S*)-2-aminobutanol production in strains expressing *M*. *marinum* CAR (MmCAR). In those strains, we determined approximately 1.5-fold more (*S*)-2-aminobutanol under pH-adjusted growth conditions. Moreover, the strain expressing CAR from *Nocardia iowensis* (NiCAR) produced (*S*)-2-aminobutanol in quantifiable amounts when buffered to pH 7 (Fig. 4a, green bars). Integration of all pathway genes, including the CAR from *M. marinum* and its accessory protein SFP, resulted in an approximate threefold higher level of (*S*)-2-aminobutanol production compared with episomal expression of the pathway genes (Fig. 4b, green bars). Even without (*S*)-2-ABA supplementation, production levels of (*S*)-2-aminobutanol reached 0.45 ± 0.03 mg/L (Fig. 4b, red bars).

### Increasing titers of (*S*)-2-aminobutyric acid

**Fig. 5:**
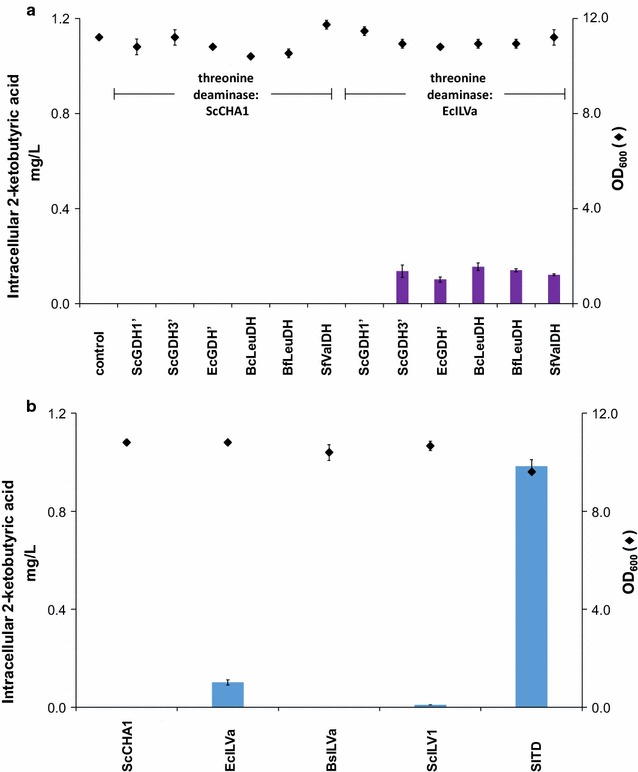
Intracellular accumulation of 2-ketobutyric acid upon 24 h of growth in selective SC medium. **a** Two different enzymes for the first step of the (*S*)-2-aminobutyric acid pathway were expressed (ScCHA1 and EcILVa) in combination with either one of six heterologous enzymes for the second step of the pathway. In case ScCHA1 was expressed or yeasts were not engineered (control), accumulation of 2-ketobutyric acid was below the limit of quantification. **b** EcGDH’ for the second step of the pathway is expressed in combination with either one of five different threonine deaminating enzymes. *Diamonds* indicate OD_600_ after 24 h of growth (all data: mean ± SD, n = 3)

As (*S*)-2-aminobutyric acid is critical for production of (*S*)-2-aminobutanol, we focused on boosting the biosynthesis of this compound. To investigate the kinetic coupling of the two heterologous enzymatic steps of the (*S*)-2-ABA pathway, we first determined the amount of 2-ketobutyric acid in strains previously analyzed for (*S*)-2-ABA production (Fig. 2). Combining ScCHA1 with any of the six enzymes for the second step of the pathway does not lead to detectable amounts of 2-ketobutyric acid. In contrast, combining EcILVa with any of the six enzymes for the second enzymatic step resulted in 2-ketobutyric acid accumulation in the range of 0.1 mg/L, except for the combination EcILVa and ScGDH1′ where no 2-ketobutyric acid could be detected (Fig. 5a). The highest accumulation of 2-ketobutyric acid (0.98 ± 0.03 mg/L) was observed with threonine deaminase (SlTD) from *S. lycopersicum* combined with EcGDH’ (Fig. 5b). We concluded that in the case of the *S. lycopersicum* threonine deaminase, a more efficient enzymatic coupling between threonine deaminase and glutamate dehydrogenase may improve conversion of l-threonine to (*S*)-2-ABA.

### Optimizing enzymatic coupling

**Fig. 6:**
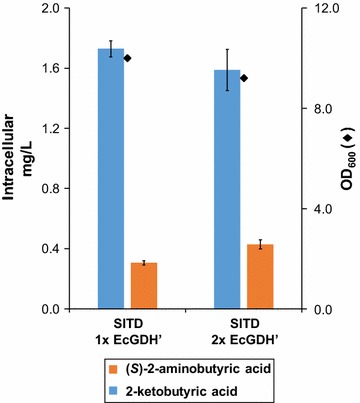
Intracellular accumulation of (*S*)-2-aminobutyric acid and 2-ketobutyric acid in *S. cerevisiae*, expressing threonine deaminase SlTD, and one or two copies of mutated glutamate dehydrogenase EcGDH’. Yeasts expressing SlTD were co-transformed with either one or two copies of the mutated glutamate dehydrogenase derived from *E. coli*. Concentrations of (*S*)-2-aminobutyric acid (*orange*) and 2-ketobutyric acid (*blue*) were analyzed in the yeast pellets upon 24 h of growth in batch culture. *Diamonds* indicate OD_600_ after 24 h of growth (all data: mean ± SD, n = 3)

Next, we sought to optimize biosynthetic titers through improved enzymatic coupling. First, we introduced an additional copy of a mutated glutamate dehydrogenase from *E. coli* and combined it with the deaminase derived from *S. lycopersicum*. This genetic modification lead to 1.4-fold more (*S*)-2-ABA (Fig. 6, orange bars). The concentration of 2-ketobutyric acid (Fig. 6, blue bars) is slightly diminished in the strain expressing two copies of EcGDH’, indicating that: (1) increasing the enzymatic capacity of the second enzyme indeed improves coupling of the two heterologous enzymes, and (2) additional optimization of coupling may further enhance (*S*)-2-ABA production.

### Increasing (*S*)-2-aminobutyric acid production by feeding l-threonine

l-Threonine is the starting molecule for enzymatic synthesis of (*S*)-2-aminobutyric acid. The concentration of l-threonine in our synthetic complete (SC) medium is 76 mg/L. When measured intracellularly upon 24 h of shake flask growth, the intracellular concentration of l-threonine is about 1.5–3 mg/L l-threonine (not shown). We hypothesized that elevating the l-threonine concentration may well influence (*S*)-2-ABA production.

**Fig. 7:**
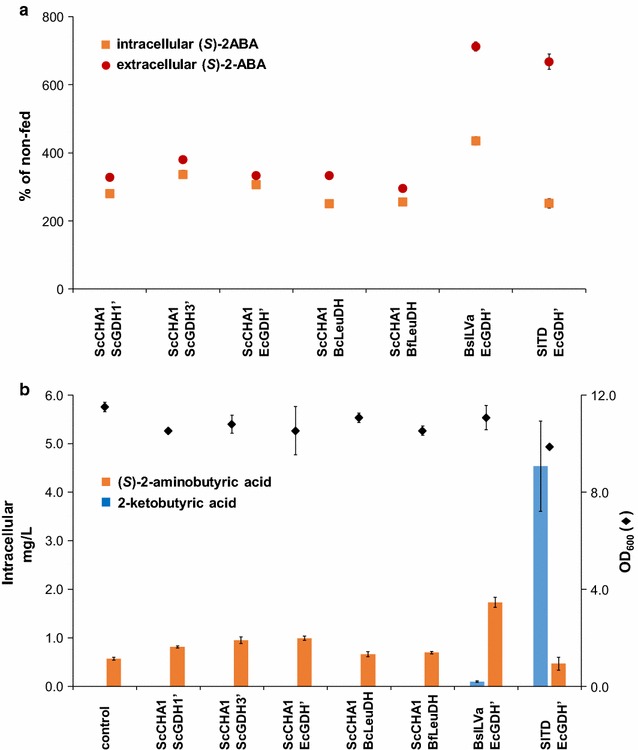
Impact of l-threonine feeding on accumulation of (*S*)-2-aminobutyric acid and 2-ketobutyric acid. Yeasts were transformed with the gene combinations indicated on the* x-axis*’. Clones were grown for 24 h in selective medium fed with 1.0 g/L l-threonine. **a** (*S*)-2-Aminobutyric acid amounts accumulating in l-threonine-fed yeasts, shown as a percentage of non-fed yeasts; *orange squares*: intracellular (*S*)-2-aminobutyric acid, *red circle*: extracellular (*S*)-2-aminobutyric acid. **b** Absolute intracellular concentrations of (*S*)-2-aminobutyric acid (*orange bars*) and 2-ketobutyric acid (*blue bars*) in l-threonine fed yeasts. *Diamonds* in **b** indicate OD_600_ after 24 h of growth (all data: mean ± SD, n = 3)

The addition of 1.0 g/L of l-threonine resulted in intracellular concentrations of l-threonine of 13–15 mg/L (8 mg/L for the combination SlTD and EcGDH’) which led to 250 to 435% intracellular (*S*)-2-ABA production in all evaluated strains (Fig. 7a). The effect is even more pronounced for extracellular concentrations of (*S*)-2-ABA (up 300–710%) in threonine–fed strains (for absolute values see Additional file 1: Figure S2). Obviously l-threonine feeding impacts not only on endogenous accumulation of (*S*)-2-ABA but also on secretion of the compound. Additional l-threonine feeding leads to a significant elevation of intracellular 2-ketobutyric acid in the strain expressing *S. lycopersicum* threonine deaminase (4.6-fold, in comparison to the non-fed conditions). The higher amounts of 2-ketobutyric acid did not translate into higher amounts of (*S*)-2-ABA, again suggesting a bottleneck at the second enzymatic step. The absolute concentration of (*S*)-2-ABA in the best producer strain (BsILVa + EcGDH’) reached 1.73 ± 0.10 mg/L (Fig. 7b).

### Upregulation of l-threonine metabolism in *S. cerevisiae* by mutating *HOM3* or deletion of *GLY1*

#### Mutation of aspartate kinase *HOM3*

*Saccharomyces cerevisiae* can synthesize l-threonine via a pathway that starts with the amino acid l-aspartate, and involves five enzymes (HOM3, HOM2, HOM6, THR1, and THR4) that assemble the amino acid via the four intermediates l-4-aspartyl-phosphate, l-aspartate-4-semialdehyde, l-homoserine, and O-phospho-l-homoserine [29]. It is known that l-threonine inhibits aspartate kinase activity in *S. cerevisiae* [30] and several mutant strains that overproduce l-threonine have been isolated which all contained a mutation in the aspartate kinase gene *HOM3* that led to insensitivity towards feedback inhibition [31, 32].

**Fig. 8:**
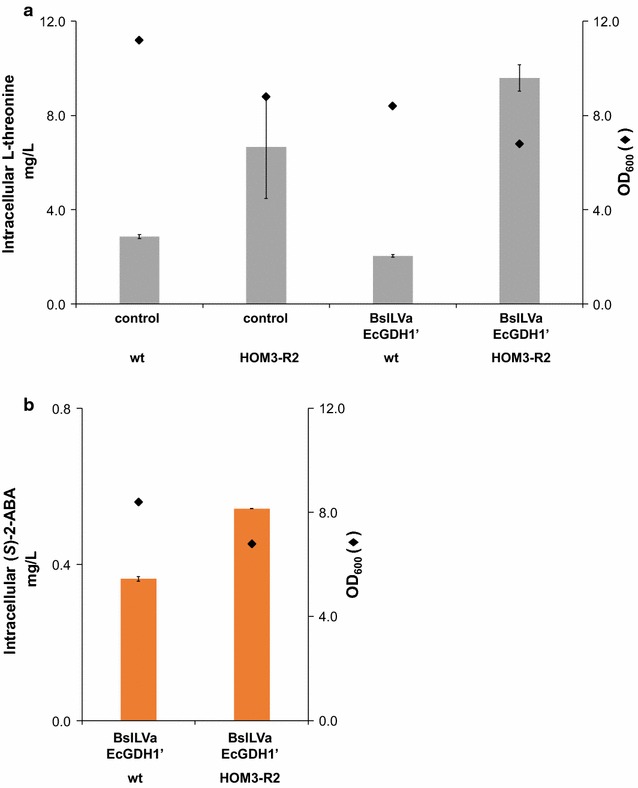
**a** Intracellular l-threonine concentrations in yeasts expressing *HOM3*-R2. Yeasts, either engineered for (*S*)-2-aminobutyric acid production (BsILVa + EcGDH’), or non-engineered yeasts, were transformed with a 2µ plasmid harboring the sequence encoding for mutated *HOM3* (*HOM3*-R2) or they were transformed with an empty plasmid (wt). Intracellular l-threonine concentrations were analyzed as described in “Methods”. *Diamonds* indicate OD_600_ after 24 h of growth (all data: mean ± SD, n = 3). **b** Intracellular (*S*)-2-aminobutyric acid concentrations in yeasts expressing *HOM3*-R2. Yeasts engineered for (*S*)-2-aminobutyric acid production (BsILVa + EcGDH’) were transformed with a 2µ plasmid harboring the sequence encoding for mutated *HOM3* (*HOM3*-R2) or they were transformed with an empty plasmid (wt). Intracellular (*S*)-2-aminobutyric acid concentrations were analyzed as described in “Methods”. *Diamonds* indicate OD_600_ after 24 h of growth (all data: mean ± SD, n = 3)

We introduced such a mutated *HOM3* gene via a 2μ plasmid into our yeast strains. This led to 2.3-fold higher l-threonine concentrations in strains not expressing the (*S*)-2-ABA pathway genes, and to a 4.7-fold increase in strains expressing the pathway genes (Fig. 8a). The higher amount of intracellular l-threonine also boosted the concentration of intracellular (*S*)-2-ABA 1.5-fold (Fig. 8b). As the boost in (*S*)-2-ABA production upon expression of the mutated *HOM3* variant is accompanied by an impaired growth, the relative accumulation of (*S*)-2-ABA compared to the number of yeast cells is even higher.

#### Deletion of threonine aldolase (*GLY1*)

**Fig. 9:**
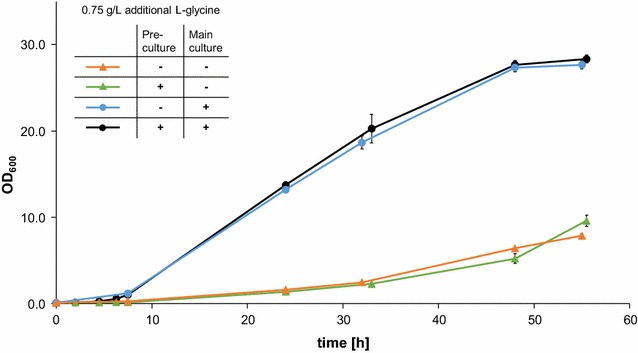
Growth of Δ*gly1* deletion strain. Δ*gly1* deletion strains were grown in selective medium either containing additional glycine (0.75 g/L l-glycine), or devoid of any additional glycine. *Black* pre-culture and main culture contain l-glycine, *blue* pre-culture contains no l-glycine, main culture contains l-glycine, *green* pre-culture contains l-glycine, main culture contains no l-glycine, *orange* no l-glycine in pre-culture nor in main culture

We tried another strategy to enhance the concentration of l-threonine, the deletion of the gene encoding for l-threonine aldolase *GLY1*. This enzyme irreversibly converts l-threonine into l-glycine [33]. We deleted the entire open reading frame of the unique *GLY1* locus on chromosome V. Our Δ*gly*1 deletion strain was viable, albeit with impaired growth characteristics as also described previously [34, 35]. Routinely we grew the Δ*gly1* deletion strain in medium that was supplied with 0.75 g/L l-glycine in the medium. It was not critical whether the pre-culture was prepared in glycine-containing medium or not. In both cases, the OD_600_ after 55 h of growth in shake flasks reached values above 27. However, omitting l-glycine in the main culture led to a massive growth retardation (OD_600_ below 7), as shown in Fig. 9. Therefore, transformation of plasmids into the Δ*gly1* deletion strain and growth of the strains was routinely done in l-glycine supplemented medium. *GLY1* wildtype and Δ*gly1* deleted strains were transformed with two enzyme combinations, BsILVa + EcGDH’ and SlTD + EcGDH’.

**Fig. 10:**
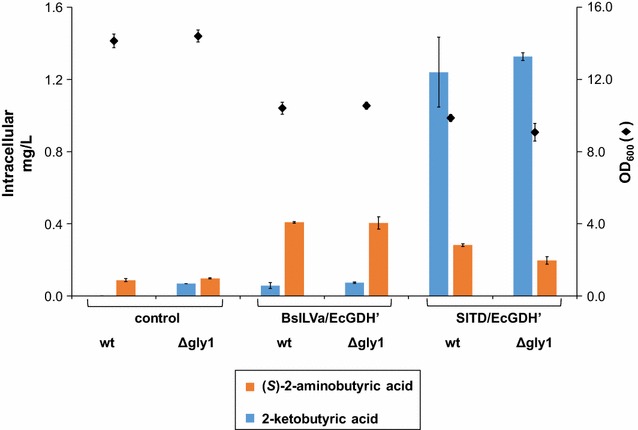
Intracellular accumulation of 2-ketobutyric acid and (*S*)-2-aminobutyric acid in *S. cerevisiae* not expressing *GLY1*. Wildtype and Δ*gly1* deletion yeast strains were either engineered for (*S*)-2-aminobutyric acid production (BsILVa + EcGDH’ or SlTD + EcGDH’), or they contained the empty plasmids (control). Concentrations of (*S*)-2-aminobutyric acid (*orange bars*) and 2-ketobutyric acid (*blue bars*) were analyzed in the yeast pellets upon 24 h of growth in batch culture. *Diamonds* indicate OD_600_ after 24 h of growth (all data: mean ± SD, n = 3)

The deletion of *GLY1* does not seem to have an effect on the amount of both extra- and intracellular l-threonine (Additional file 1: Figure S3), except for the SlTD + EcGDH’ strain, which produces roughly two times more l-threonine than the other strains. The elevated concentration of l-threonine in the SlTD + EcGDH’ Δ*gly1* deletion strain does not translate into an elevated concentration of 2-ketobutyric acid or (*S*)-2-aminobutyric acid (Fig. 10). In strains expressing SlTD + EcGDH’ (both *GLY1* wildtype and Δ*gly1* deletion strain) the amount of 2-ketobutyric acid exceeds 1.2 mg/L. The concentration of (*S*)-2-ABA does not exceed 0.3 mg/L in those strains. The concentration of 2-ketobutyric acid is significantly lower in the BsILVa + EcGDH’ strains, suggesting tight coupling of 2-ketobutyric acid generation and subsequent conversion to (*S*)-2-aminobutyric acid, as observed previously.

## Discussion

In this study, we describe: (1) the production of enantiopure (*S*)-2-aminobutyric acid in *Saccharomyces cerevisiae* by expressing two heterologous genes that convert l-threonine to (*S*)-2-aminobutyric acid; (2) the prolongation of the pathway from (*S*)-2-aminobutyric acid to (*S*)-2-aminobutanol by expressing three additional heterologous genes. Both compounds are of considerable interest due to their potential use for the production of the chiral anti-epileptic drugs levetiracetam and brivaracetam, as well as for the production of the chiral anti-tuberculosis drug ethambutol. The latter one figures on the WHO list of essential medicines.

### Pathway to (*S*)-2-aminobutyric acid

We show that expression of *B. subtilis* threonine deaminase, combined with expression of a mutated form of *E. coli* glutamate dehydrogenase leads to the production of 0.40 ± 0.02 mg/L of (*S*)-2-aminobutyric acid in shake flask-grown *S*. *cerevisiae* cells. The higher production in *E. coli* achieved by Zhang and co-workers [26] is perhaps due to the special properties of the *E. coli* strain employed, which can produce 8 g/L l-threonine from 30 g/L glucose. Nevertheless, we rationalize that yeast is indeed a superior production host for (*S*)-2-aminobutanol production, as it displays higher robustness and considerable tolerance against harsh fermentation conditions. Yeast is also more resistant towards exposure to (*S*)-2-aminobutanol (Additional file 1: Figure S4). Moreover, the fermentation of yeasts is easily implemented into existing ethanol productions plants, and there are no issues with phage contamination. *S. cerevisiae* is classified as GRAS (“generally regarded as safe”) organism; thereby further facilitating industrial scale up.

We have explored a variety of strategies to increase the yield of (*S*)-2-ABA production in our yeast strains; first by evaluating the effect of adding a second copy of mutated glutamate dehydrogenase. When analyzing intracellular 2-ketobutyric acid concentrations we realized that in yeasts containing the enzyme combinations threonine deaminase from *S. lycopersicum* and mutated glutamate dehydrogenase from *E. coli,* a considerable amount of 2-ketobutyric acid (0.98 ± 0.03 mg/L) was not converted to (*S*)-2-aminobutyric acid. This may be due to the relatively low K_m_ value of this heterologous l-threonine deaminase (SlTD: 0.25 mM, [36]). In contrast, the deaminase from *E. coli* has a K_m_ of 11 mM (if AMP is present, otherwise it is 91 mM) [37], and the one from *B. subtilis* is 9.6 mM [38]. The amount of l-threonine generated in the strains after 24 h of growth in shake flasks reaches approximately 2 mg/L (~0.017 mM intracellular l-threonine). This low intracellular l-threonine concentration can be more efficiently converted to 2-ketobutyric acid by SlTD as this deaminase has a lower K_m_ than the other threonine deaminases.

It is expected that the obvious massive increase in production of 2-ketobutyric acid by SlTD would lead to a substantial elevation of (*S*)-2-ABA accumulation, especially taking into account the relatively high K_m_ (8.4 mM) of EcGDH’ [26]. As this was not the case, the enzymatic capacity of *E. coli* glutamate dehydrogenase was enhanced by expressing a second copy of this enzyme in the yeast. The modification elevated the amount of (*S*)-2-aminobutyric acid, but a considerable amount of 2-ketobutyric acid remained. This indicates that production of (*S*)-2-ABA may not be massively tunable, probably due to substrate inhibition of the *E. coli* dehydrogenase [39]. This hypothesis is further substantiated by the fact that in threonine fed strains expressing SlTD and EcGDH’, 2-ketobutyric acid production was elevated to above 4.54 ± 0.93 mg/L, without increasing intracellular (*S*)-2-aminobutyric acid accumulation.

Feeding with l-threonine elevated intracellular (*S*)-2-ABA accumulation in strains expressing the enzyme combination BsILVa and EcGDH’ more than fourfold compared to non-fed strains.

The conversion yield of l-threonine to (*S*)-2-ABA (no additional l-threonine) was 36% compared to 22% with additional l-threonine in the medium, indicating that l-threonine is consumed by enzymatic and/or efflux reactions triggered by the elevated intracellular l-threonine concentration.

Interestingly, in contrast to the non-fed strains, most of the de novo (*S*)-2-ABA was found in the supernatant, indicating that (*S*)-2-ABA was either actively secreted, or passively diffused. l-Threonine strongly induces the broad-specificity amino acid permease AGP1 [40, 41], suggesting that this permease may not only mediate uptake of l-threonine, but also increase (*S*)-2-ABA efflux. Another putative mediator of (*S*)-2-ABA efflux could be the internal-membrane transporter AQR1. This transporter is involved in excretion of excess amino acids by exocytosis [42], suggesting that it may also secrete (*S*)-2-ABA.

As feeding with l-threonine significantly boosted (*S*)-2-ABA production, we hypothesized that upregulation of threonine anabolism may enhance generation of (*S*)-2-ABA in our engineered strains.

The G425D mutation of aspartate kinase HOM3, previously described to abrogate feedback-inhibition, led to a significant boost of both intracellular l-threonine and (*S*)-2-aminobutyric acid. Recently, Mülleder et al. analyzed the amino acid metabolome of *S. cerevisiae* upon systematic gene deletions [43]. They found that deletion of FPR1 (a peptidyl-prolyl cis–trans isomerase) enhanced intracellular l-threonine by around 2.5-fold. This increase is in good accordance with our HOM3 mutant, indicating that FPR1 may indeed regulate feedback inhibition of HOM3 as previously suggested [31, 44]. l-Threonine and (*S*)-2-aminobutyric acid were also detected at higher extracellular levels upon expression of the mutated form of HOM3. Those data indicate that the endogenous l-threonine supply is indeed critical for (*S*)-2-ABA production.

The l-threonine aldolase GLY1 of *S. cerevisiae* catalyzes the cleavage of l-threonine into l-glycine and acetaldehyde. In contrast to expectations, no elevated l-threonine levels were detected in Δ*gly1* deletion strains. The comparable concentrations of l-threonine accumulated in wild type and Δ*gly1* deletion strains led us to conclude that feedback inhibitory mechanisms are active e.g. aspartate kinase HOM3. Our Δ*gly1* deletion strains showed strongly impaired growth characteristics compared to the wildtype strains (four to fivefold lower). These growth characteristics are similar to the ones previously reported [34], suggesting that GLY1 is important for l-glycine synthesis.

### Pathway to (*S*)-2-aminobutanol

Here, we report production of the non-natural compound (*S*)-2-aminobutanol in *S. cerevisiae*. The highest production levels were observed in strains expressing (apart from the enzymes forming the (*S*)-2-aminobutyric acid pathway) *M. marinum* carboxylic acid reductase and an aldehyde reductase from *E. coli*. The level of 0.42 ± 0.07 mg/L (*S*)-2-aminobutanol could be reached by buffering the growth medium to pH 7. Valli and co-workers [45] demonstrated previously that yeast cells grown at pH 7 are viable and adjust their endogenous pH accordingly. Furthermore, CARs work predominantly at basic pH conditions [46]. We hypothesized therefore that adjusting the culture medium to pH 7 would increase intracellular pH and lead to elevated activity of heterologous CARs. Our results support this hypothesis. Without pH adjustment of the surrounding medium, the extracellular pH drops to pH 2.5 in stationary phase [45] that would correspond to an intracellular pH around 5, which is not suitable for CAR activity [46].

Interestingly, integration of the entire pathway into a single position of chromosome XI significantly enhanced production of (*S*)-2-aminobutanol, even though promoters and terminators of the heterologous genes were identical to those used for episomal expression. Growth of strains expressing the plasmid-encoded pathway was however significantly lower than yeast strains expressing the genome-integrated pathway. These results indicate that plasmid maintenance is perhaps at the expense of heterologous pathway expression and product generation. However, strains expressing the pathway also displayed retarded growth. One explanation for impaired cellular growth and proliferation are the reductive modifications of intracellular fatty acids or reduction of fatty acids that form parts of membrane lipids by CARs [46, 47]. Ultimately, compartmentalization of the heterologous pathway might be a valuable approach for efficient orthologous production of this non-natural chiral compound.

Previous studies have demonstrated that substrates with an amine group at the α-position are scarcely accepted by carboxylic acid reductases [48], suggesting that production of (*S*)-2-aminobutanol may be further boosted by optimizing the substrate binding site of the carboxylic acid reductase.

### Co-factor supply in the pathway

Threonine deaminases are pyridoxal-5′-phosphate (PLP) dependent, with the PLP co-factor remaining in the active site and being regenerated during the reaction cycle [49]. In *S. cerevisiae* PLP levels are elevated when the yeasts are grown under thiamine deficient conditions as the synthesis of PLP is inhibited by the presence of thiamine [50]. Enhanced conversion in whole-cell biocatalysis experiments employing PLP-dependent ω-transaminases and addition of PLP or preceding growth in thiamine-deficient medium have been shown [50, 51].

Most of the enzymes in the pathway to (*S*)-2-aminobutanol require NADPH. Elevated NADPH supply should boost production of (*S*)-2-aminobutyric acid and (*S*)-2-aminobutanol, as shown previously for several other cofactor-dependent enzymatic reactions [52–55]. NADPH is generated predominantly by the pentose phosphate pathway. A recent study has shown that overexpression of glucose-6-phosphate dehydrogenase ZWF1, the enzyme that catalyzes the first step of the pentose phosphate pathway, or overexpression of NADH kinase POS5 leads to a higher of NADPH-dependent heterologous production of β-carotene in *S. cerevisiae* [56]. In future studies, we will use the described approaches to enhance the efficiency of the heterologous pathway.

## Conclusions

We have successfully assembled a heterologous metabolic pathway in *S. cerevisiae* to produce the non-proteinogenic amino acid (*S*)-2-aminobutyric acid and the purely synthetic compound (*S*)-2-aminobutanol. To our knowledge (*S*)-2-aminobutanol has never previously been produced biosynthetically. Our studies expand the spectrum of synthetic biology towards the biosynthesis of e.g. pharmaceutical compounds by entirely novel and sustainable means.

## Methods

### Chemicals and media

l-threonine, 2-ketobutyric acid, (*R*)- and (*S*)-aminobutyric acid, (*R*) and (*S*)-2-aminobutanol were bought from Sigma-Aldrich Chemie AG (Steinheim, Germany). Yeast Extract Peptone (YPD) medium with 20 g/L glucose was used for growth of the wildtype strain prior to transformation and both bought from Sigma-Aldrich Chemie AG. Synthetic complete (SC) drop out medium (Formedium LTD, Hustanton, England) with 6.7 g/L yeast nitrogen base without amino acids and ammonium sulfate (Sigma-Aldrich Chemie AG), and 20 g/L glucose (Sigma-Aldrich Chemie AG) was used for pre- and main cultures. LB medium for growth of *E. coli* was supplied from Carl Roth GmbH + Co. KG (Karlsruhe, Germany).

### Primers, genes, and strains

Primers used in this study were supplied by Microsynth AG (Balgach, Switzerland) and are shown in Additional file 1: Table S1. Genes used in this study are listed in Table 1. *Saccharomyces cerevisiae* strains generated throughout this study are listed in Table 2. All yeast strains were stored in 25% glycerol at −80 °C.

**Table 1:** Expression cassettes and plasmids

Feature	Plasmid type	ORF origin	Description	Uniprot number	Reference
pGPD1-ScCHA1-tCYC1	Entry vector	*Saccharomyces cerevisiae*	Threonine deaminase	P25379	This study
pGPD1-EcILVa-tCYC1	Entry vector	*Escherichia coli*	Threonine deaminase	P04968	[26]
pGPD1-SlTD-tCYC1	Entry vector	*Solanum lycopersicum*	Threonine deaminase	P25306	[61]
pGPD1-ScILV1-tCYC1	Entry vector	*Saccharomyces cerevisiae*	Threonine deaminase	P00927	This study
pGPD1-BsILVa-tCYC1	Entry vector	*Bacillus subtilis*	Threonine deaminase	P37946	[26]
pPGK1-ScGDH1′-tADH2	Entry vector	*Saccharomyces cerevisiae*	Glutamate dehydrogenase (K74V/T177S)	P07262	This study
pPGK1-ScGDH3′-tADH2	Entry vector	*Saccharomyces cerevisiae*	Glutamate dehydrogenase (K75V/T178S)	P39708	This study
pPGK1-EcGDH’-tADH2	Entry vector	*Escherichia coli*	Glutamate dehydrogenase (K92V/T195S)	P00370	[26]
pPGK1-BcLeuDH-tADH2	Entry vector	*Bacillus cereus*	Leucine dehydrogenase	C2RD20	[8]
pPGK1-BfLeuDH-tADH2	Entry vector	*Bacillus flexus*	Leucine dehydrogenase	A0A0L1MCT3	This study
pPGK1-SfValDH-tADH2	Entry vector	*Streptomyces fradiae*	Valine dehydrogenase	P40176	[27]
pTEF1-NiCAR-tENO2	Entry vector	*Nocardia iowensis*	Carboxylic acid reductase	Q6RKB1	[28]
pTEF1-NfCAR-tENO2	Entry vector	*Nocardia farcinica*	Carboxylic acid reductase	Q5YY80	This study
pTEF1-MmCAR-tENO2	Entry vector	*Mycobacterium marinum*	Carboxylic acid reductase	B2HN69	[46]
pTEF1-MsCAR-tENO2	Entry vector	*Mycobacterium smegmatis*	Carboxylic acid reductase	L0IYJ8	[48]
pTEF2-BsSFP-tPGI1	Entry vector	*Bacillus subtilis*	Phosphopantetheinyl transferase	P39135	[46]
pTEF2-MsPPTase-tPGI1	Entry vector	*Mycobacterium smegmatis*	Phosphopantetheinyl transferase	L0ITG8	[48]
pPDC1-EcALR-tFBA1	Entry vector	*Escherichia coli*	Aldehyde reductase	A0A094VUC2	This study
pURA3-ScURA3-tURA3	Entry vector	*Saccharomyces cerevisiae*	Uracil marker	–	–
ARS/CEN	Entry vector	*Saccharomyces cerevisiae*	Origin of replication	–	–
pPYK1-tTEF1	2 micron	*Saccharomyces cerevisiae*	Vector only	–	–
pTEF2-tPGI1	ARS/CEN	*Saccharomyces cerevisiae*	Vector only	–	–
pPYK1-ScHOM3-R2-tTEF1	2 micron	*Saccharomyces cerevisiae*	Aspartate kinase (G425D)	P10869	[31, 32]
pTEF2-EcGDH’-tPGI1	ARS/CEN	*Escherichia coli*	Glutamate dehydrogenase (K92 V/T195S)	P00370	[26]

**Table 2:** Strains

Strains	Description	Selection marker	Replication origin/integration site
EVST20590	Contains expression cassettes of ScCHA1 and ScGDH1′	URA3	ARS/CEN
EVST20591	Contains expression cassettes of ScCHA1 and ScGDH3′	URA3	ARS/CEN
EVST20592	Contains expression cassettes of ScCHA1 and EcGDH’	URA3	ARS/CEN
EVST20879	Contains expression cassettes of ScCHA1 and BcLeuDH	URA3	ARS/CEN
EVST20880	Contains expression cassettes of ScCHA1 and BfLeuDH	URA3	ARS/CEN
EVST20881	Contains expression cassettes of ScGDH1′ and EcILVa	URA3	ARS/CEN
EVST20882	Contains expression cassettes of ScGDH3′ and EcILVa	URA3	ARS/CEN
EVST20883	Contains expression cassettes of EcGDH’ and EcILVa	URA3	ARS/CEN
EVST20884	Contains expression cassettes of EcILVa and BcLeuDH	URA3	ARS/CEN
EVST20885	Contains expression cassettes of EcILVa and BfLeuDH	URA3	ARS/CEN
EVST21351	Contains expression cassettes of ScCHA1 and SfValDH	URA3	ARS/CEN
EVST21352	Contains expression cassettes of EcILVa and SfValDH	URA3	ARS/CEN
EVST21478	Contains expression cassettes of ScCHA1, EcGDH’, NiCAR, EcALR, and BsSFP	URA3	ARS/CEN
EVST21479	Contains expression cassettes of ScCHA1, EcGDH’, NfCAR, EcALR, and BsSFP	URA3	ARS/CEN
EVST21542	Control strain with empty entry vectors	URA3	ARS/CEN
EVST22605	Contains expression cassettes of BsILVa and EcGDH’	URA3	ARS/CEN
EVST22606	Contains expression cassettes of ScILV1 and EcGDH’	URA3	ARS/CEN
EVST22608	Contains expression cassettes of SlTD and EcGDH’	URA3	ARS/CEN
EVST22609	Contains expression cassette of ScHOM3-R2	LEU2	2 micron
EVST22610	Contains expression cassettes of BsILVa, EcGDH’, and ScHOM3-R2	URA3 (BsILVa, EcGDH’), LEU2 (ScHOM3-R2)	ARS/CEN (BsILVa, EcGDH’) 2 micron (ScHOM3-R2)
EVST22615	Contains expression cassettes of EcILVa, EcGDH’, MsCAR, MsPPTase, and EcALR	URA3	ARS/CEN
EVST22837	Contains expression cassette of SlTD and two EcGDH’ expression cassettes	URA3, HIS3	ARS/CEN both
EVST22857	Deletion of *GLY1* ORF	–	–
EVST23406	Deletion of *GLY1* ORF Contains expression cassettes of BsILVa and EcGDH’	URA3	ARS/CEN
EVST23407	Deletion of *GLY1* ORF Contains expression cassettes of SlTD and EcGDH’	URA3	ARS/CEN
EVST24185	Contains expression cassettes of BsILVa, EcGDH’, MmCAR, EcALR, and BsSFP	URA3	ARS/CEN
EVST25556	Control strain with empty plasmid	LEU2	2 micron
EVST25557	Contains expression cassettes of BsILVa, EcGDH’, and empty plasmid	URA3 (BsILVa, EcGDH’), LEU2	ARS/CEN (BsILVa, EcGDH’) 2 micron (empty plasmid)
EVST25635	Contains expression cassettes of SlTD, EcGDH’, and empty plasmid	URA3 (BsILVa, EcGDH’), HIS3	ARS/CEN both
EVST27022	Contains expression cassettes of BsILVa, EcGDH’, MmCAR, EcALR, and BsSFP	URA3	integrated into Chromosome XI-2

### Molecular biology

Plasmid DNA was prepared using the ZR Plasmid Miniprep-Classic Kit (Zymo Research Corp, Irvine, CA, USA). Competent *E. coli* NEB 10-β cells (New England Biolabs, Herts, United Kingdom) were used for all cloning steps. Restriction enzymes were supplied from New England Biolabs (Herts, United Kingdom). T4 ligase was from Agilent (Santa Clara, CA, USA), and thermostable DNA polymerase iProof™ High-Fidelity was purchased from BioRad (Hercules, CA, USA). dNTPs were from Roche Diagnostics GmbH (Mannheim, Germany). PCR reactions were performed in a FlexCycler^2^ (Analytik Jena AG, Jena, Germany) using the appropriate cycling conditions. PCR products were purified using the DNA Clean & Concentrator-5 Kit from Zymo Research Corp (Irvine, CA, USA). Accuracy of genetic constructs was verified by sequencing (Microsynth AG, Balgach, Switzerland).

### Plasmid construction, gene deletion, and integration

Most of the genes used in this study were supplied in plasmids as yeast-codon optimized versions of the original genes (GeneArt, Thermo Fisher Scientific, Waltham, MA, USA). Genes were released from the plasmids, supplied by the manufacturer, by *Hin*dIII/*Sac*II digestions prior to ligation into our pre-cut entry vectors. Our entry vectors contain different combinations of promoters and terminators, which are flanked by 60 bp homologous regions (“linkers”) that enable the assembly of multiple expression cassettes in vivo by homologous recombination [57, 58]. Accessory entry vectors dispose of autonomously replicating sequences, centromere regions, and auxotrophic markers as described in Eichenberger et al. [59]. One-pot digestion of entry vectors by *Asc*I releases the expression cassettes composed of (1) promoter-gene-terminator flanked by 60 bp linkers at the 5′ and 3′ end; (2) selection marker URA3 flanked by 60 bp linkers at the 5′ and 3′ end; and (3) replication origin ARS/CEN flanked by 60 bp linkers at the 5′ and 3′ end); or, for integration, two 560 bp homologous regions flanked by 60 bp linkers at the 5′ and 3′ sites. Released fragments were then transformed into the strains as described in the next paragraph.

Amplification of the plasmids containing the carboxylic acid reductase from *Nocardia farcinica* has been reported to be not feasible in *E. coli* (personal communication Esben H. Hansen). Therefore, the gene was ordered in two fragments, containing a 60 bp homologous region. The entire open reading frame was fused in vivo in yeast. Correct assembly was confirmed by PCR amplification and subsequent sequencing.

*HOM3* was amplified from yeast genomic DNA in three parts. The three sequence stretches were fused by overlap extension PCR thereby removing the *Hin*dIII site and mutating glycine 425 to aspartic acid. The resulting entire PCR product was cut with *Hin*dIII and *Sac*II, and inserted into a 2μ yeast expression plasmid.

The ORF encoding GLY1 was deleted by transformation of a PCR product encoding the marker LEU2 flanked by homologous regions upstream and downstream of the genomic *GLY1* ORF. Successful deletion was confirmed by colony PCR.

The five pathway genes (BsILVa, EcGDH’, MmCAR, BsSFP, EcALR) were integrated by homologous recombination of the same cassettes as used for the episomal expression plasmid (described before), except that the centromere region was exchanged with the homologous region on chromosome XI-2 (noncoding region between FAT3 and MTR2).

### Transformation and cell growth

Yeast transformation was performed using the lithium acetate method described in [60]. Transformed clones were grown on agar plates prepared with selective SC drop out medium. Single colonies from the plates were inoculated into 3 mL of selective liquid SC medium and grown overnight at 30 °C in a shaker (160 rpm). Main cultures (25 mL in 250 mL shake flasks) for production of (*S*)-2-ABA were inoculated at a starting OD_600_ of 0.1, and kept on the shaker at 30 °C for 24–72 h. The main cultures for (*S*)-2-aminobutanol were carried out in 24 deepwell plates (OD 0.1, 2.5 mL, 72 h, 30 °C, 300 rpm; Porvair, Norfolk, UK).

### Extraction

Cell suspensions in 10 mL of growth medium were harvested by centrifugation at 4000 rpm for 5 min. One millilitre of the supernatant was removed for analysis. The remaining cell pellet was resuspended in 1 mL water and added to a 2 mL screw cap tube containing 500 µL glass beads (0.5 mm, Huberlab, Aesch, Switzerland). Cell lysis was performed by a precellys 24 bead beater (Bertin technologies, Aix-en-Provence Cedex, France) for 3 cycles for 45 s with 60 s break. Cell debris was removed by centrifugation (14,000 rpm, 5 min), and the supernatant was subjected to analyses.

### Analyses

Stock solutions of (*R*)- and (*S*)-2-aminobutyric acid, l-threonine, 2-ketobutyric acid, and 2-aminobutanol (1 g/L) were prepared in water. Calibration standards of those compounds were prepared in the range of 31 μg/L–8 mg/L in water/acetonitrile 85:15.

Pellet extracts (150 μL) or supernatants (150 μL) were diluted into 850 μL of acetonitrile (in order to precipitate protein remainings), and centrifuged prior to injection.

### Threonine, 2-ketobutyric acid, 2-aminobutyric acid, and 2-aminobutanol analysis

Samples were injected into an Acquity UPLC-TQD (Waters), equipped with an Aquity UPLC BEH Amide 1.7 μm 2.1 × 100 mm (Waters) column with two mobile phases. A: water with 10 mM ammonium formate and 0.15% formic acid, and B: acetonitrile with 2 mM ammonium formate and 0.05% formic acid. Separation was performed with a gradient ranging from 85 to 82% of mobile phase B for 3 min then to 60% B in 1 min at a flow rate of 0.6 mL/min. Then a washing step at 60% B for 1 min, followed by a reconditioning step of 1 min at 85% B, was carried out. 2-Ketobutyric acid was detected with SIR mode following the mass 101.1 in ESI- with a cone voltage of 20 V. 2-Aminobutanol, 2-aminobutyric acid, and l-threonine were detected with MRM mode with the transitions 90.1 > 55, 104.1 > 58, 120 > 74 in ESI+ , cone voltages of 18, 16 and 16 V and collision voltages of 12 V for all compounds.

### 2-Aminobutyric acid chiral analysis

Samples were injected on an Aquity UPLC-TQD (Waters) equipped with an Astec Chirobiotic T 250 × 4.6 mm column (Sigma) with water/methanol/formic acid 30:70:0.02 isocratic mobile phase and 1 mL/min flow during 16 min. (*R*)- and (*S*)-aminobutyric acid were detected with MRM mode with the transition 104.1 > 58.0 in ESI+ , a cone voltage of 16 V and collision voltage of 12 V (Additional file 1: Figure S1).

## Additional file

**Additional file 1.** Additional figures and table.

## Abbreviations

(*S*)-2-ABA: (*S*)-2-aminobutyric acid (synonyms: α-aminobutyric acid, l-homoalanine)
ScCHA1: *Saccharomyces cerevisiae* threonine deaminase CHA1
EcILVa: *Escherichia coli* threonine deaminase ILVa
BsILVa: *Bacillus subtilis* threonine deaminase ILVa
ScILVa: *Saccharomyces cerevisiae* threonine deaminase ILVa
SlTD: *Solanum lycopersicum* threonine deaminase
ScGDH1′: *Saccharomyces cerevisiae* glutamate dehydrogenase 1 (K74V/T177S)
ScGDH3′: *Saccharomyces cerevisiae* glutamate dehydrogenase 3 (K75V/T178S)
EcGDH’: *Escherichia coli* glutamate dehydrogenase (K92V/T195S)
BcLeuDH: *Bacillus cereus* leucine dehydrogenase
BfLeuDH: *Bacillus flexus* leucine dehydrogenase
SfValDH: *Streptomyces fradiae* valine dehydrogenase
NiCAR: *Nocardia iowensis* carboxylic acid reductase
NfCAR: *Nocardia farcinica* carboxylic acid reductase
MsCAR: *Mycobacterium smegmatis* carboxylic acid reductase
MmCAR: *Mycobacterium marinum* carboxylic acid reductase
HOM3-R2: *Saccharomyces cerevisiae* aspartate kinase (G425D)
EcALR: *Escherichia coli* aldehyde reductase
ScGLY1: *Saccharomyces cerevisiae* threonine aldolase
BsSFP: *Bacillus subtilis* phosphopantetheinyl transferase
ORF: open reading frame
