# Supplementary material for: Production of (S)-2-aminobutyric acid and (S)-2-aminobutanol in Saccharomyces cerevisiae
Source: Microb Cell Fact. 2017 Mar 23;16:51. doi: 10.1186/s12934-017-0667-z (PMC5364695; doi:10.1186/s12934-017-0667-z)
Supplement: Supplementary file 1 — Additional file 1. Additional figures and table. [file 12934_2017_667_MOESM1_ESM.docx]

**Supplementary Material**

**Production of (*S*)-2-aminobutyric acid and (*S*)-2-aminobutanol in *Saccharomyces cerevisiae***

Nora Weber*, Anaëlle Hatsch, Ludivine Labagnere, and Harald Heider

**NW:** noraw@evolva.com

**AH**: anaelleh@evolva.com

**LL**: ludivinel@evolva.com

**HH:** haraldh@evolva.com

Evolva SA, Duggingerstrasse 23, 4153 Reinach, Switzerland

^*^ Corresponding author. Phone: +41 61 485 20 93, Email: noraw@evolva.com

Tables

Table S1. Primers

| **Name** | **Sequence 5’→3’** |
| --- | --- |
| EVPR11580 (NfCAR seq) | AATGAGCAGCTGGAGACA |
| EVPR11847 (NfCAR seq) | TGATACCACTGTTAGAAGACC |
| EVPR11066 (NiCAR InFusion) | TAATTACAAAAAGCTTAAAATGGCTGTTGATTCACCAGATGAG |
| EVPR11067 (NiCAR InFusion) | AGTTAAAAGCACTCCGCGGTTATAACAATTGTAACAATTCCAAATCAGAAACATACTTATCAATCAATG |
| EVPR11071 (CgPPTase InFusion) | TTAACTAAACAAGCTTAAAATGCTGGATGAGTCTTTGTTTCCAAA |
| EVPR11072 (CgPPTase InFusion) | AAGAGCGATTTGTCCGCGGTCAAGTCACTGCAGTCGCAGC |
| EV859 (NiCAR seq) | CTTTCGATGACCTCCCATTG |
| EV2675 (NiCAR seq) | AGTGTTCTAAACTATGATG |
| EVPR11068 (NiCAR seq) | CTCCAGAAAGATTGGTTGT |
| EVPR11069 (NiCAR seq) | TTGGGTTATTTCAGAACTGAC |
| EVPR11070 (NiCAR seq) | CTCTAACTTGTTACATGAG |
| EVPR11183 (NiCAR seq) | TATGCAATAGACAGCACGA |
| EV2668 (CgPPTase seq) | CTCTCTATCTATTCTACTTG |
| EV2669 (CgPPTase seq) | TCCTTTGCATTACGTACAT |
| EVPR12700 (*HOM3*-R2 cloning) | TGGAAGCTTATGCCAATGGATTTCCAACCT |
| EVPR12701 (*HOM3*-R2 cloning) | TACTCCAGAAGAAGCTaCTGAATTAACATAT |
| EVPR12702 (*HOM3*-R2 cloning) | ATATGTTAATTCAGtAGCTTCTTCTGGAGTA |
| EVPR12703 (*HOM3*-R2 cloning) | TGAAACAATACATCGaCATTGCTGGTACCAT |
| EVPR12704 (*HOM3*-R2 cloning) | ATGGTACCAGCAATGtCGATGTATTGTTTCA |
| EVPR12705 (*HOM3*-R2 cloning) | ACGCCGCGGTTAAATTCCAAGTCTTTTCAATTGTTC |
| EV2666 (*HOM3*-R2 seq) | TTCCAGTTATATCATGGTC |
| EV2667 (*HOM3*-R2 seq) | CTATACAAATGACAAGTTCT |
| EVPR12706 (*GLY1* deletion) | ttacactcacttttactcccgcacacaaacgcaaacataaacacaCCAGCTGAAGCTTCGTACGC |
| EVPR12707 (*GLY1* deletion) | ctaaaacaaaaaccctaacaatacacatgatgcaactggaacgcaGCATAGGCCACTAGTGGATCTG |
| EVPR12708 (*GLY1* colony PCR) | agtaagctaagcttaacta |
| EVPR12709 (*GLY1* colony PCR) | gttgttgaagtgaagata |

**Figure S1**

**Chiral analysis of (*S*)-2-aminobutyric acid.** Chromatograms of pure (*S*)-2-aminobutyric acid (A), pure (*R*)-2-aminobutyric acid (B), yeast pellet extract (C) and yeast pellet extract spiked with (*R*)-2-aminobutyric acid (D) obtained by LC-MS analysis on chiral column.


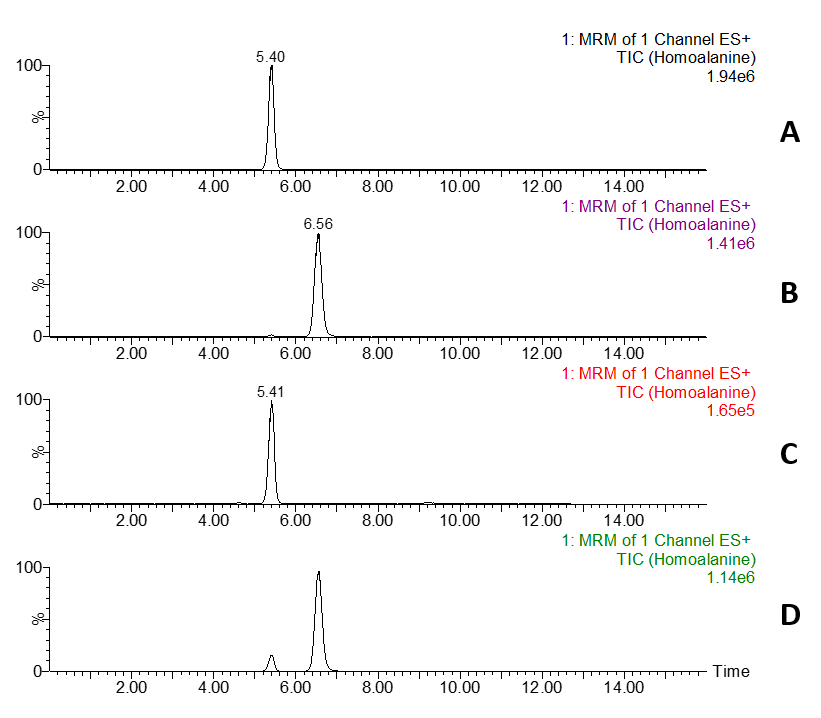


**Figure S2**

**Extracellular accumulation of (*S*)-2-aminobutyric acid (panels A+B, and orange bars in panel C) and 2-ketobutyrate (blue bar in panel C) in *S. cerevisiae* expressing different enzyme combinations.** A) Two different threonine deaminases (orange bars: ScCHA1, green bars: EcILVa) for the first step of the (*S*)-2-aminobutyric acid pathway were expressed in combination with either one of six heterologous enzymes for the second step of the pathway (**no** additional threonine). B) EcGDH’ for the second step of the pathway was expressed in combination with either one of five different threonine deaminases (**no** additional threonine). C) Three different enzymes for the first step of the (*S*)-2-aminobutyric acid pathway were expressed in combination with either one of five heterologous enzymes for the second step of the pathway (**1.0 g/L additional L-threonine**). Orange bars: (*S*)-2-aminobutyric acid, blue bars: 2-ketobutyrate. Diamonds indicate OD_600_ after 24 h of growth (all data: mean ± SD, n=3).

**
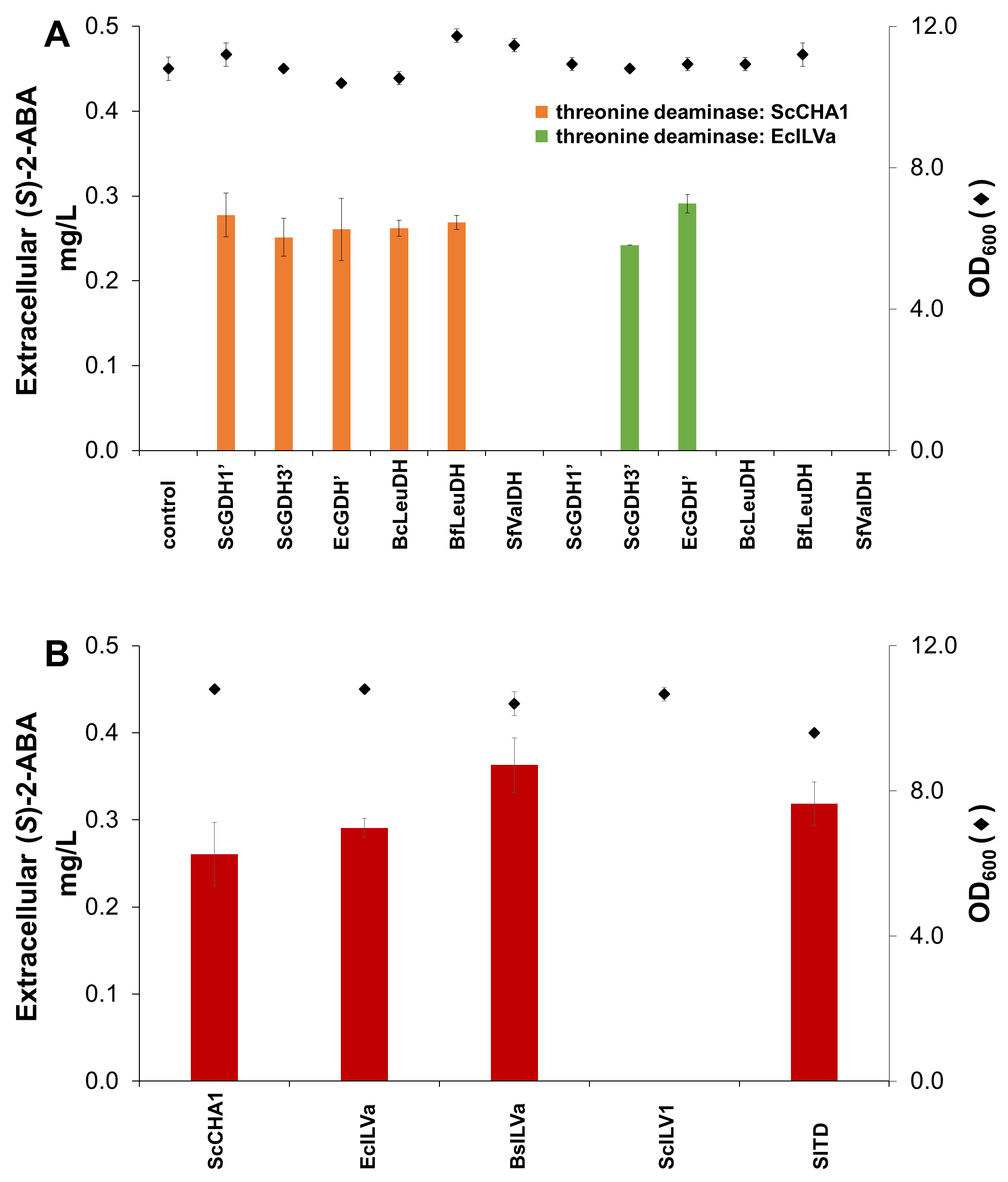

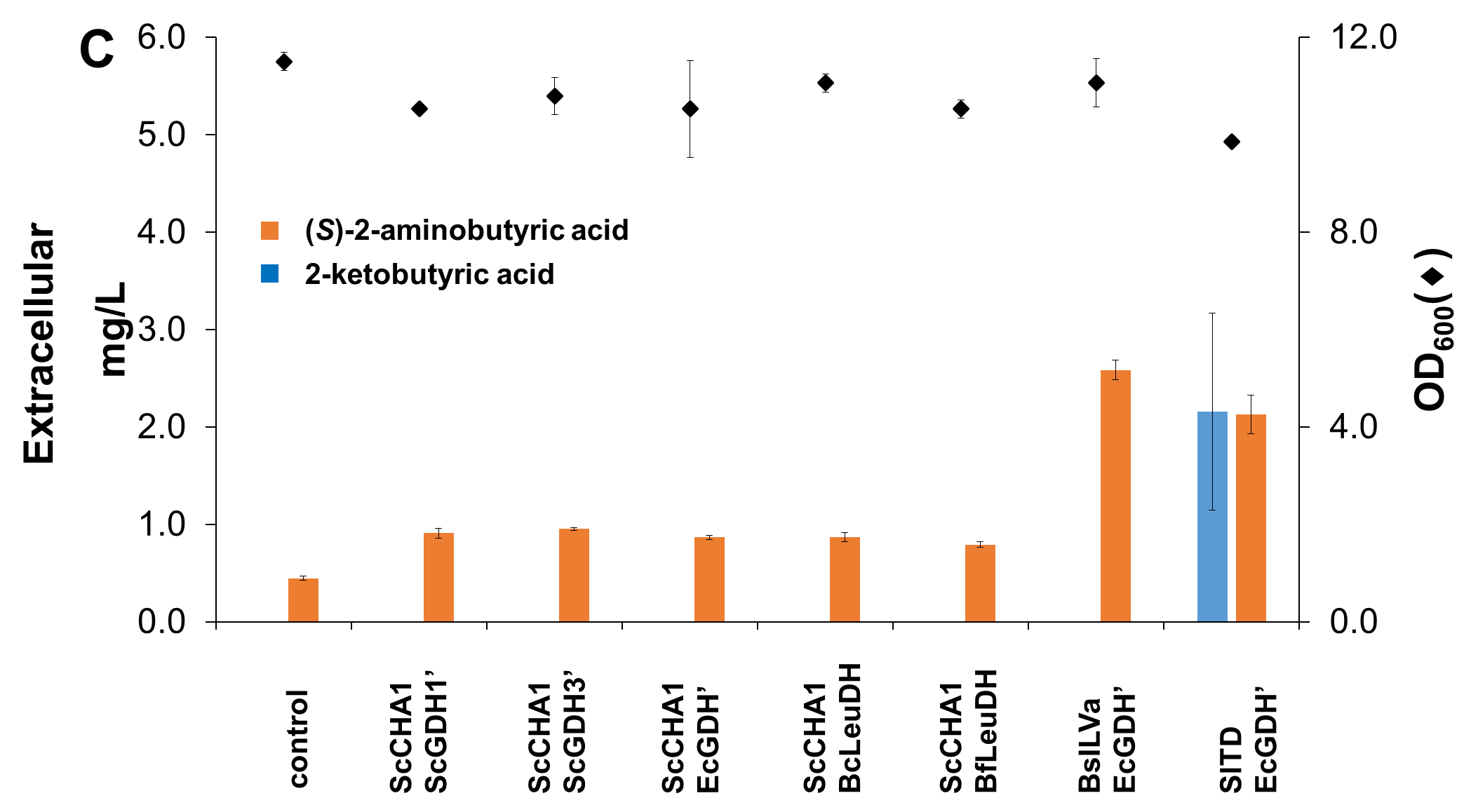
**

**Figure S3**

**Accumulation of intracellular and extracellular L-threonine in wild type and Δ*gly1* deletion strains.** Wild type and Δ*gly1* deletion strains were transformed with EcGDH’ in combination with either BsILVa or SlTD, or they were transformed with empty plasmids (wt and Δgly1). Gray bars: intracellular L-threonine, red bars: extracellular L-threonine. Diamonds indicate OD_600_ after 24 h of growth (all data: mean ± SD, n=3).


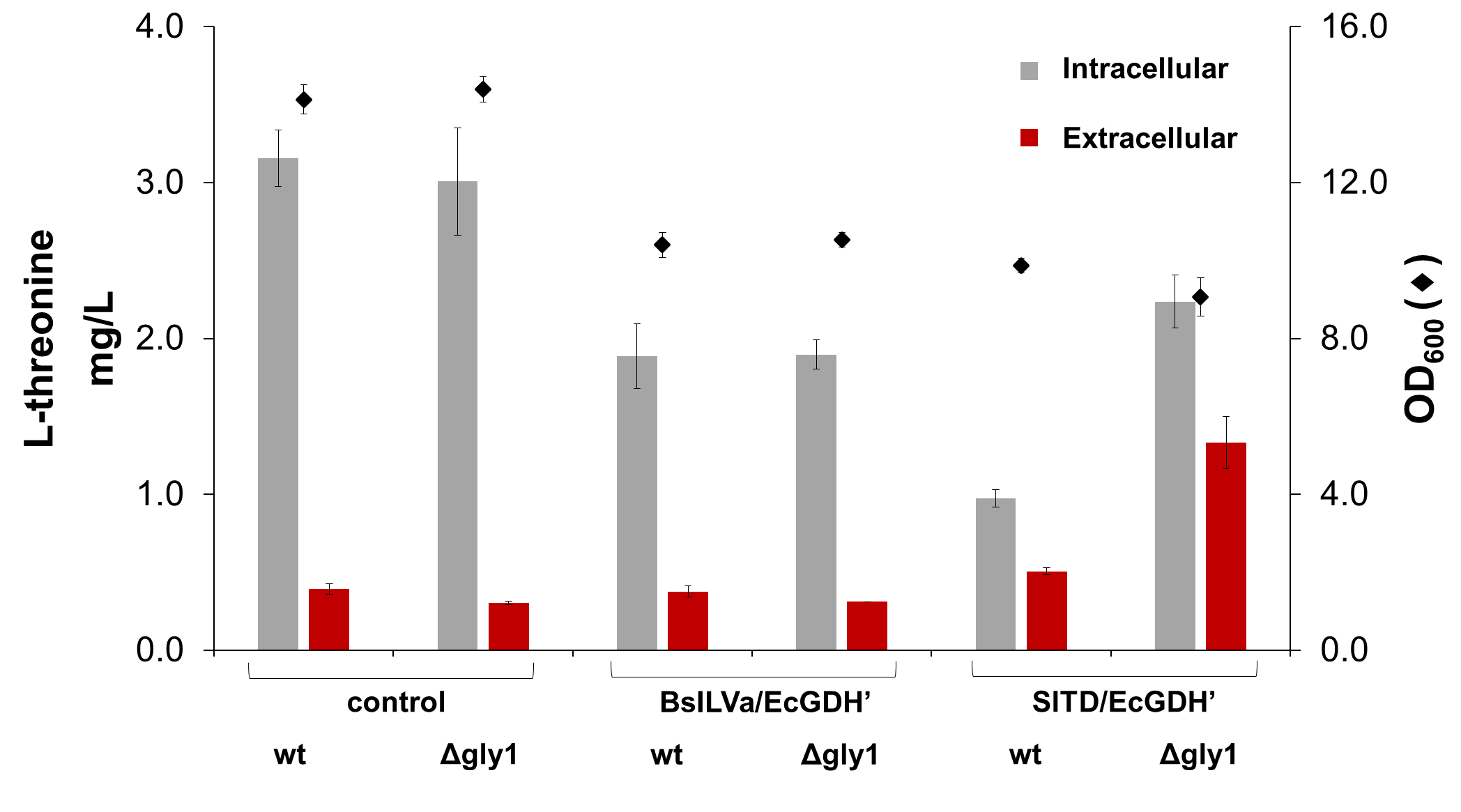


**Figure S4**

**Growth of *E. coli* and *S. cerevisiae* in the presence of (*S*)-2-aminobutanol at different concentrations.** Both microorganisms were first grown in 3 mL LB (*E.coli*) or YPD (yeast) overnight in shake flasks. Then they were inoculated at OD 0.1 into individual wells of a BioLector (m2p-labs GmbH, Baesweiler, Germany) plate, containing either 0.8 mL LB or YPD medium with 0 – 1.0 g/L (*S*)-2-aminobutanol. Individual cultures were measured in triplicates until they reached stationary phase (7 h for *E. coli* and 13 h for *S. cerevisiae*).

**
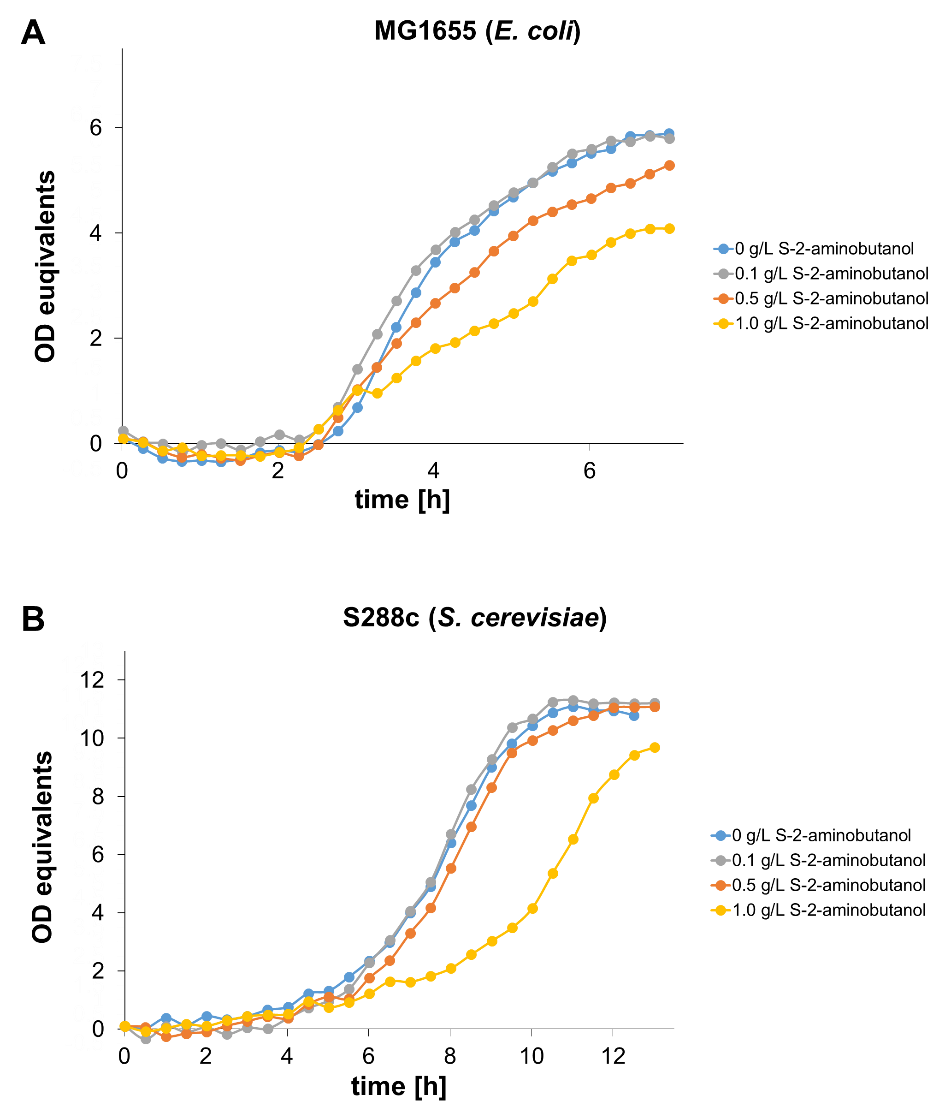
**
